# Atypical Presentation of COVID-19 Leading to ARDS

**DOI:** 10.1155/2020/1070249

**Published:** 2020-07-02

**Authors:** Aquino Williams, Lance Alquran, Ummi Khan, Gabriela Bambrick-Santoyo, Roveena Goveas

**Affiliations:** Department of Medicine, Hackensack Meridian Health Mountainside Medical Center, Montclair, New Jersey 07028, USA

## Abstract

A 60-year-old male was brought into the emergency department by EMS after he was found unresponsive by his neighbors. He was initially admitted to the hospital for chronic obstructive pulmonary disease exacerbation secondary to pneumonia. However, due to a sudden, rapidly progressing course of events, the patient was evaluated for COVID-19.

## 1. Introduction

An acute respiratory disease caused by a new strand of coronavirus (SARS-CoV-2), which was first identified in Wuhan, Hubei Province, China, quickly developed into a global pandemic. Common signs associated with coronavirus disease 2019 (COVID-19) include fever, cough, and shortness of breath. The World Health Organization (WHO) suggests having a high level of suspicion for patients with severe or acute respiratory infection/illness associated with a fever, especially those with exposure to risk factors. These exposures include individuals who have had contact with confirmed or probable cases of COVID-19 within 14 days of symptom onset [1]. Furthermore, the Centers for Disease Control and Prevention strongly encourages testing for other respiratory illnesses including influenza [2]. Currently, no specific treatment for the virus exists, and the current goals of management include supportive care, including support of vital organ functions.

## 2. Case Report

A male in his sixties with a past medical history of non-insulin-dependent type 2 diabetes mellitus, chronic obstructive pulmonary disease without the use of home oxygen, hypertension, and hypothyroidism was initially brought to the hospital by EMS after being found unresponsive at home by neighbors. Prior to the collapse, the patient reported having symptoms of dizziness, cough, fatigue, and an inability to keep his balance for one day. However, he did not have any of the typical suspicious symptoms, which correlate with COVID-19. He denied having dyspnea, myalgia, gastrointestinal symptoms, travel history, sick contacts, headaches, visual changes, loss of consciousness, palpations, or chest pain.

**Figure 1:**
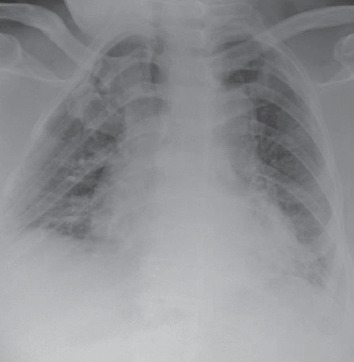
Cardiac silhouette is enlarged and vascular calcifications are present; increased central vascular lung markings; and no pulmonary edema, lobar consolidation, effusion, or pneumothorax.

Upon admission, the patient was awake, alert, and oriented. His vitals were a temperature of 101°F, blood pressure of 137/82 mmHg, heart rate of 72 bpm, respiratory rate of 18 breaths per minute, and pulse oxygen saturation of 99% via a 6 L nasal cannula. His skin was dry and pale. Rale was heard on bilateral lower lung fields. Initial testing revealed that creatine kinase levels were greater than 5,000 U/L. The patient also had mild elevations in aspartate aminotransferase and alanine aminotransferase. Furthermore, lactic acid and leukocyte count were within normal range. There were no premature neutrophils (bands) present, and rapid flu testing was negative. Admission chest X-ray was unremarkable for acute changes when compared to studies from previous admission (Figure 1).

**Figure 2:**
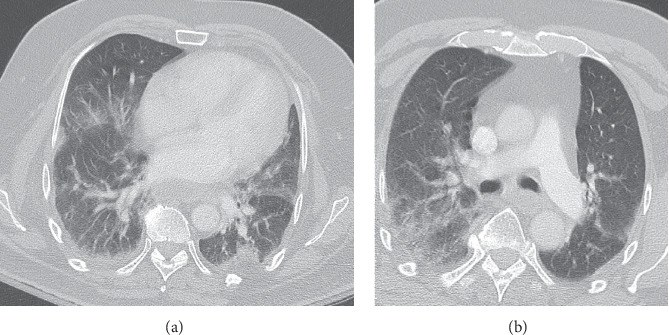
(a) Subpleural and peripheral interstitial infiltrate [3–5] which is worrisome for coronavirus or other viral or atypical infections. (b) Computed tomography angiography showed negative for pulmonary embolus but showed signs of subpleural and peripheral interstitial infiltrate suggestive of COVID-19 vs. other viral infections [3–5]. However, prior chest X-ray on admission was negative for any acute changes when compared to previous studies.

The patient was admitted to the medical floor and was started on ceftriaxone and azithromycin for presumed pneumonia vs. bronchitis leading to a presumed chronic obstructive pulmonary disease exacerbation. The patient had a one-time read of fever on admission; vitals and labs were relatively stable during the first forty-eight hours. On day 2 of admission, the patient remained stable and asymptomatic with no changes on physical exam. However, fifty-two hours after admission, he became febrile with a temperature of 101.7°F, and his oxygen saturation declined to 88% on room air and 92% via a 6 L nasal cannula. Notably, he was found to be diaphoretic but able to talk in full sentences. Physical exam revealed new wheezing sounds in the bilateral lung bases. Given his deteriorating conditions, it was decided to test the patient for COVID-19 given the rapid spread of the virus nationwide. Prior to this point, the patient did not have any of the typical criteria that were identified in current papers or guidelines from the Centers for Disease Control and Prevention, besides a low-grade fever. At this point, necessary samples were taken (respiratory viral panel, COVID-19, rapid strep, and throat culture) and appropriate isolation was initiated. Arterial blood gas was obtained while the patient was on room air, which showed 45% partial pressure of oxygen (PaO_2_) and 84% saturation. Due to concerns for additional complications including differential such as a pulmonary embolism, the patient was sent for a CT angiography of the chest (Figures 2(a) and 2(b)), which was negative for pulmonary embolism but did show findings consistent with COVID-19-positive patients [3–5].

**Figure 3:**
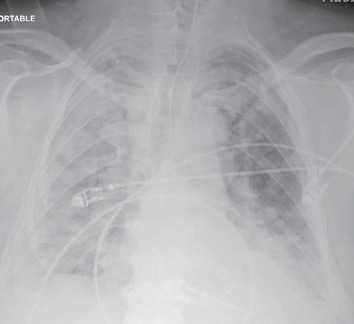
A significant gross increase in diffuse bilateral infiltrates with some sparing of the right upper lobe, which had worsened when compared to previous chest X-rays.

Due to an increased risk of aerosolization while on BiPAP, the patient was placed on 50% high-flow nasal cannula (HFNC) which improved his saturations to 98% and PaO_2_ to 102 mmHg, while still maintaining a low threshold for possible intubation. He maintained a stable respiratory status for several days, including ambulation without becoming symptomatic/desaturating. Respiratory status was stable for approximately four days while the patient was on HFNC, with saturation maintaining above 92%, without signs of acute distress. However, the patient continued to spike fevers intermittently, with a maximum temperature of 103.2°F. Oxygen requirements on HFNC decreased on day 3 from 50% to 35%, where he was able to maintain his SaO_2_ above 94%. The SARS-CoV-2 test was positive. On day 5 and after this clinical improvement, he developed acute hypoxemic respiratory failure requiring intubation and transfer to the intensive care unit (ICU). He was found to have acute respiratory distress syndrome (ARDS) secondary to COVID-19 (Figure 3). The ventilation setting was adjusted for the management of ARDS, which improved oxygenation; however, the patient remained hypercapnic. Noticeably, the patient's blood pressure declined and required pressors. Plaquenil and Kaletra were initiated 6 days after admission.

On day 10, which was approximately four days into his ICU stay, the patient developed cardiac arrest and later died.

## 3. Discussion

Coronaviruses are described as enveloped, positive single-stranded RNA viruses, which have the ability to infect humans as well as a broad range of animals [6]. There are four known subfamilies of the coronavirus: alpha-, beta-, delta-, and gamma-coronaviruses [7]. The beta-coronaviruses can cause severe disease in humans. SARS-CoV-2 is part of the B lineage of beta-coronaviruses, which causes COVID-19 (coronavirus disease 2019) [7]. The current outbreak is of the novel coronavirus SARS-CoV-2, which originated in the Hubei Province of the People's Republic of China and has since spread globally [7]. SARS-CoV-2 infects alveolar epithelial cells via receptor-mediated endocytosis using the angiotensin-converting enzyme II receptors in the lungs [8]. SARS-CoV-2 has a wide variety of initial symptoms. Patients can range from asymptomatic infections to only having symptoms of the gastrointestinal tract [9]. Approximately 14% (13.8%, *n* = 44,672) of patients develop severe disease, which requires hospitalization and oxygen support, and around 5% require intensive care unit management [10]. In patients who become symptomatic, clinical manifestations of COVID-19 can consist of fever, nasal congestion, fatigue, cough, and other typical signs of respiratory infections [11]. The symptoms of infected patients usually present within one week [11]. An accurate percentage of infected patients who are asymptomatic has not yet been possible to be assessed [7]. In observational studies to date, the mean incubation period is five days, with a median incubation period of three days [11] (interquartile range 2 to 7). Pneumonia usually occurs around the second to third week in patients who are symptomatic [9]. Notable signs of viral pneumonia are arterial blood gas abnormalities and decreased oxygen saturations, as well as changes seen on imaging [9, 11]. Common abnormities seen on imaging (*n* = 101) include ground-glass opacities (86.1%), combined ground-glass opacities and consolidation (64.4%), bilateral lung involvement (82.2%), as well as alveolar exudates, and interlobular involvement [4, 9]. Another common finding is leukopenia on complete blood counts (82.1%, *n* = 1,099) [9, 11]. COVID-19 is considered a mild illness when patients have uncomplicated upper respiratory tract symptoms. Adults who develop pneumonia but have no signs of severe disease (respiratory rates greater than 30 and SpO_2_ equal to or less than 93% on room air) do not require oxygen supplementation [12]. Management of patients with mild disease does not require hospitalization. However, isolation is mandatory to contain virus transmission [12]. Patients with mild disease should receive symptomatic relief. They should also be counseled on the potential of developing severe disease and of seeking urgent care in case symptoms worsen. This is also a recommendation for physicians as displayed by this patient who seemingly improved before rapidly declining. Oxygen therapy should be given immediately to patients with respiratory distress, hypoxemia, and shock. The target oxygen saturation should be >94% [12]. Patients will need close monitoring for signs of clinical deterioration. Management of critically ill patients with COVID-19 is considered when patients develop acute respiratory distress syndrome (ARDS). If ARDS develops, intubation should be performed.

The initial tidal volume ranging between 4 and 8 mL/kg of predicted body weight and lower plateau pressure <30 cm H_2_O are recommended. Prone ventilation is recommended for 12–16 hours per day for patients with severe ARDS [12]. Conservative fluid management is recommended when there is no tissue hypoperfusion. For moderate to severe ADRS, high PEEP and a PaO_2_/FiO_2_ less than 150 mmHg should also be used [12]. Adjunctive therapy with corticosteroids is not recommended, as a systemic review of observational studies administered to patients with SARS in 2002-2003 reported no survival benefit and possible harms [13]. A recent small French open-label nonrandomized clinical trial showed treating confirmed COVID-19 patients with hydroxychloroquine significantly reduced viral load, and the effect was reinforced by adding azithromycin [14]. However, more recent studies have shown this treatment to be potentially harmful, as it is potentially associated with mortality due to cardiac arrhythmias as an associated adverse event, and thus cardiac monitoring is advised [15, 16]. Therefore, the application of such therapy must be carefully considered based on the patient's clinical presentation, as well as the setting in which they are being managed [15, 17].

## 4. Conclusion

Upon reflection, it was notable that our patient's arterial blood gas and CT scan were similar to another patient. Both showed hypoxemia, suggested by an alveolar-arterial gradient, and had similar CT findings. This case illustrates that our patient did not have significant symptoms or high-risk factors for COVID and thus was not tested on admission due to his stable respiratory condition. The uniqueness of this presentation lies in the syncopal presentation along with the absence of associated symptoms or risk factors at the initial time of presentation to the hospital. However, his condition deteriorated leading to testing for SARS-CoV-2 which was positive. Notably, it seemed that this patient had begun to improve; however, he had a sudden, unexpected rapid decline, which indicates the possible need for continued treatment and close monitoring throughout the course of the disease. Later, during his stay, the patient was started on Plaquenil and lopinavir/ritonavir, which arguably could have been started earlier; however, at the time of patient admission, there was no clear indication in terms of management present at that time.
